# A case report of spontaneous rupture of intracranial epidermoid cyst with dramatic increase of serum carbohydrate antigen 199: a three-year follow-up study

**DOI:** 10.1186/s12883-015-0452-8

**Published:** 2015-10-12

**Authors:** Wei Yan, Liang Xu, Qun Wu, Gao Chen, Jian-Min Zhang, Shu-Mei Wei, Yong-Jie Wang

**Affiliations:** Department of Neurosurgery, The Second Affiliated Hospital, School of Medicine, Zhejiang University, Hangzhou, 310009 Zhejiang Province P. R. China; Department of Pathology, The Second Affiliated Hospital, School of Medicine, Zhejiang University, Hangzhou, 310009 Zhejiang Province P. R. China

**Keywords:** Carbohydrate antigen 199, Intracranial epidermal cyst, Ventriculitis, Treatment

## Abstract

**Background:**

Tumor markers are widely applied in clinical practice, however, few serum markers have been found for intracranial tumors. Herein, we firstly report an intracranial epidermoid cyst case with extremely high level of serum CA 199. Furthermore, the relationship between CA 199 level and intracranial epidermoid cyst was closely followed for a long period.

**Case presentation:**

We report a case of 41-year-old man with a history of 2 months’ headache and sudden exacerbation for 3 days. Radiology examination suggested multiple lesions spreading along ventricular system. Laboratory tests showed exceeding increase of serum CA 199. The patient underwent craniotomy and continuous lumber drainage. Post-operative pathology proved a ruptured intracranial epidermoid cyst. MRI scans and serum CA 199 were closely followed up for three years.

**Conclusion:**

This case suggests an important role of serum CA 199 in the diagnosis and follow-up of intracranial epidermoid cyst. Ruptured intracranial epidermoid cyst should be considered for a sudden onset case with multiple intracranial lesions and dramatically increased serum CA 199.

## Background

Tumor markers have been widely applied in the diagnosis, differential diagnosis, prognosis and treatment evaluation of tumors [1]. The central nervous system tumors, however, lack specific and sensitive serum tumor markers, except for intracranial germ cell tumor, the diagnosis of which depends on α feto-protein and human chorionic gonadotropin [2]. Carbohydrate antigen 199 (CA 199) is originally found expressing in pancreas and bile duct, and is widely used for the diagnosis of pancreatic cancer [3]. Takeshita described two cases of intracranial epidermoid cyst with mildly to moderately elevated CA 199 level [4], and this is the only report of serum CA 199 as a potential marker of intracranial epidermoid cyst according to our literature research. Cases with high level of CA 199 and long term follow-up have never been reported. Here, we report a rare case of spontaneously ruptured intracranial epidermoid cyst with extremely high level of serum CA 199 and unusual radiological manifestation. Three-year follow-up data are obtained to study the correlation between CA 199 level and MRI characteristics, the mechanisms of which are also discussed.

## Case presentation

**Fig. 1:**
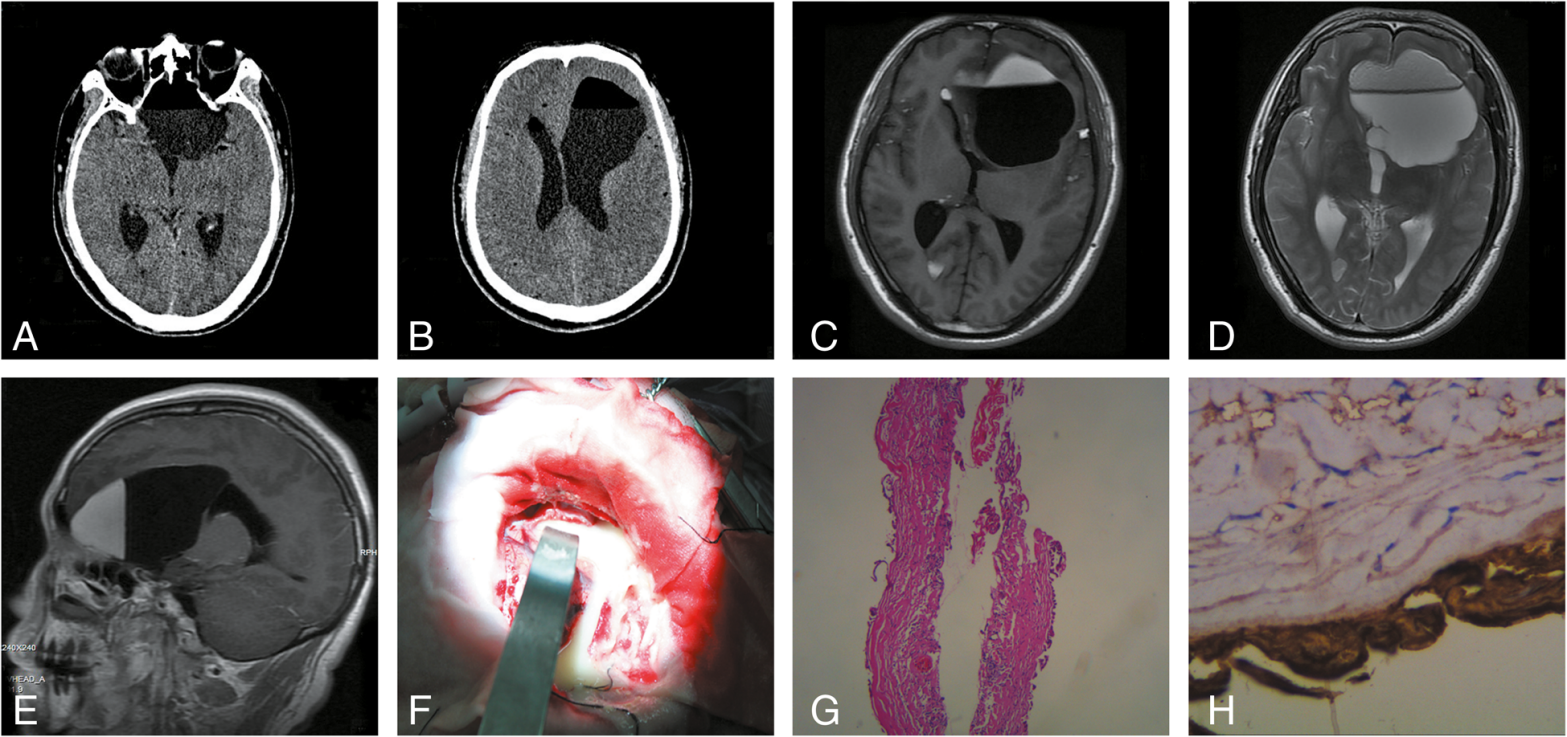
Radiological and pathological study of the patient. CT scan (**a**, **b**) before surgery displayed an irregular hypo-density mass extending from the suprasellar cistern to the anterior horn of left ventricle, with fluid-fluid level formation. MRI scan (**c**-**e**) revealed multiple hypointense lesions in the anterior horn of both ventricles and over the sulci. The major lesion in the left anterior horn displayed a low to high stratified signal on T1-weighted image (**c**), slightly high to high on T2 weighted image (**d**) and no enhancement after contrast administration (**e**). Intraoperatively, the tumor was found to be cystic in nature with a yellowish lipoid content (**f**). Hematoxylin and eosin staining (**g**, magnification × 40) of the capsule demonstrated stratified squamous epithelium supported by an outer layer of collagenous connective tissue. The immunohistochemistry (**h**, magnification × 400) of tumor capsule displayed strong positive staining of CA 199

**Fig. 2:**
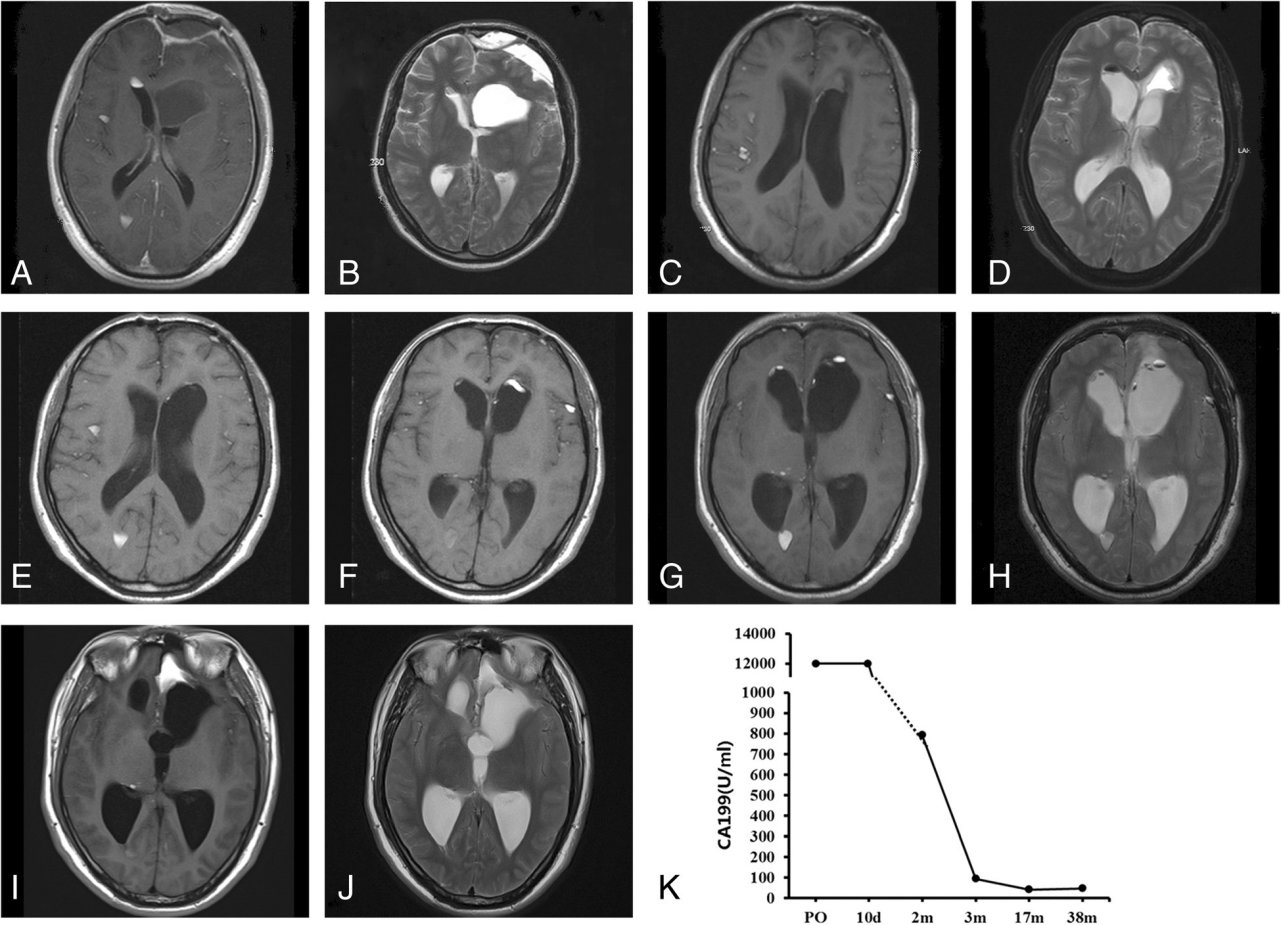
Follow-up of the patient. MRI scans half a month post-surgery (**a**, **b**), one month post-surgery (**c**, **d**), half a year post-surgery (**e**, **f**) and one and half a year post-surgery (**g**, **h**) showed that the size of residual tumor decreased gradually, which correlated with the trend of decreased value of CA 199 (**k**). While the latest MRI scan three years post-surgery (**i**, **j**) showed an enlargment of the lesion in the left anterior cranial base, consistently, the value of CA 199 raised up from 41.7U/ml to 47.0 U/ml. PO, pre-operation

A 41-year-old man was referred to us with a history of 2 months’ headache and sudden exacerbation for 3 days, accompanied by nausea, vomiting, slurred speech, blurred vision and a fever of 38.3 °C. The physical examination revealed mental confusion, rigid neck and dull light reflex of left eye. Brain computed tomography (CT) displayed a 6.7 cm × 6.9 cm × 5.5 cm irregular hypodense mass extending from the suprasellar cistern to the anterior horn of left ventricle, where a fluid-fluid level was formed, with a CT value around −110 in the superior portion and +15 in the inferior portion (Fig. 1a, b). Subsequent magnetic resonance imaging (MRI) revealed a similar cystic lesion, with low to high stratified signal on T1 weighted imaging (T1WI) and slightly high to high signal on T2 weighted imaging (T2WI). Multiple hyperintense lesions were also identified in the anterior horn of right ventricle and over the sulci on both T1WI and T2WI (Fig. 1c-e). The diagnosis was unclear preoperatively. To rule out the possibility of intracranial metastasis, common tumor biomarkers were screened, which showed elevated serum level of CA 199, carbohydrate antigen 125 (CA 125), carbohydrate antigen 242 (CA 242) and carcinoembryonic antigen (CEA). The level of CA 199 was unexpectedly higher than 12,000 U/ml. Careful history taking, physical examination and radiological study including chest and abdomen CT ruled out common cancerous and non-cancerous diseases leading to elevated CA 199. Ruptured cystic mass was considered, and therefore, craniotomy was performed. Yellowish lipoid content (Fig. 1f) in the cystic lesion was aspirated and repeatedly douched, and the capsule was subtotally resected. Continuous lumbar drainage was conducted to facilitate cerebrospinal fluid (CSF) clearance. Pathological report proved the diagnosis of intracranial epidermoid cyst (Fig. 1e). Immunohistochemistry (IHC) of lesion capsule displayed strong staining of CA 199 (Fig. 1f) and moderate staining of CA125 and CEA. A repeat MRI scan suggested subtotal elimination of lipid content (Fig. 2a, b), and tumor markers serum level remained extremely high 10 days post-operation. The patient improved quickly and was discharged without any neurological deficit two weeks after surgery. MRI scan and serum tumor markers were closely monitored after discharge (Fig. 2). Serum CEA, CA125 and CA242 levels decreased back to normal range 2 months after surgery. CA 199 level also dropped dramatically 2 months post-operation, and decreased gradually in the following period, however, still beyond upper limit. A rebound of CA 199 level was observed during the last follow-up (38 months after surgery, Fig. 2i-k), companied by an enlargement of residual mass on MRI. The patient was in good physical condition, so no interventions were performed.

## Discussion

Intracranial epidermoid cyst accounts for 0.2– 1.8 % of all intracranial neoplasms and approximately 50 % of intracranial epidermoid cyst occurred in the cerebellopontine angle, followed by parasellar region, basal cistern, sylvian, pineal region and ventricle systems [5–7]. Unless rupture taking place, it usually follows a benign clinical course [5]. Preoperational diagnosis relies solely on radiological examination, but sometimes it is difficult to be differentiated from other cystic lesions [6]. In the case presented, the patient resorted to medical help because of a ruptured intracranial epidermoid cyst with rare MRI manifestation. Most interestingly, the serum CA 199 level of this patient was extremely high. MRI and serum CA 199 level were monitored for 38 months after surgery, and special attention was paid to the correlation between them. According to our knowledge, the case reported was the first of this kind.

On CT scans, intracranial epidermoid cyst is hypodense with calcification present in 10– 25 % of cases [6]. On MRI scans, they may display low to high signal on T1WI and usually high signal on T2WI depending on the cystic contents. Fluid attenuated inversion recovery (FLAIR) and diffusion weighted imaging (DWI) can be helpful with differentiation of intracranial epidermoid cyst from other intracranial cysitc lesions. However, the diagnosis is still sometimes confused. Although rare, intracranial epidermoid cyst can rupture spontaneously. In most cases, the cholesterol crystals spread throughout the subarachnoid space and the ventricular system, presented as multiple lesions on MRI, and resulting in chemical or aseptic meningitis. In our case, the intracranial epidermoid cyst was originally situated adjacent to the anterior horn of left ventricle. Once rupture occurred, the lipid content leaked into the ventricle, and meanwhile, the CSF rushed into the cystic lesion. Since lipid did not dissolve in CSF and possessed a lower density, stratification was observed on MRI.

The treatment of ruptured intracranial epidermoid cyst includes the use of steroids and antibiotics to release the symptoms, with combination of surgical resection [8]. Sufficient irrigation with saline and hydrocortisone has been recommended during surgery [9]. The huge lesion in our case communicated with lateral ventricle and disseminated along the CSF pathway, making total resection impossible. Therefore, in addition to the strategies mentioned above, lumbar drainage was administrated to facilitate CSF clearance.

Takeshita attributed the various levels of CA 199 to the amount of secretary glands, thickness of capsule, location of intracranial epidermoid cyst and the existence of a blood brain barrier (BBB) [4]. In our case, the dramatic increase of CA 199 could be explained by the strongly positive staining of CA 199 in capsule and the damage of BBB. We speculated that the aseptic ventriculitis and meningitis secondary to lesion rupture destroyed the BBB, resulted in increased permeability of vascular endothelium. CA 199 dispersed from cystic content into ventricular system and further into the systemic circulation. Serum CA 199 kept out-of-limit 10 days after surgery, which might be attributed to the persistence of ventriculitis and long half-life of CA 199. Serum CA 199 decreased gradually two months after operation, resulting from the evacuation of cystic content and successful control of ventriculitis. 38 months’ follow-up suggested a close relativity between serum CA 199 level and MRI manifestation. However, rupture is not the only reason leading to elevated serum CA 199. The increase of the size of epidermoid cyst, as detected by MRI during last follow-up, could generate more CA 199 bearing glands and result in elevated serum CA 199 level.

The augmentation of the other tumor markers including CEA, CA 125 and CA 242 were also observed. These tumor biomarkers, together with CA 199, were all originated from epithelial tissue. Therefore, it was not strange for CEA and CA 125 to be found positively stained in IHC and elevated in serum. As for the negative finding of CA 242 in IHC study, it may be the result of sampling error, since only subtotal resection of the capsule was achived and part of the capsule was suctioned away during the surgery.

Further study of CA 199 expression in a cohort of 41 intracranial epidermoid cyst cases in our hospital demonstrated a reproducible finding in consistent with this case report (data not reported). There were totally 12 patients with CA 199 higher than the upper limit of 37U/mL. CA 199 were significantly higher in patients with intracranial epidermoid cyst than in healthy controls (48.19 ± 73.88U/mL vs 7.0 ± 4.1U/mL, *p* < 0.001). To evaluate the diagnostic value more systematically and accurately, prospective study with more cases and longer follow-up are needed. Our interesting findings in this case and the above preliminary results, together with the data from other investigators, indicate a possible role of CA 199 in the diagnosis and follow-up of intracranial epidermoid cyst.

## Conclusions

This is the first case to suggest that serum CA 199 might be a marker for diagnosis and follow-up of intracranial epidermoid cyst. For a sudden onset case with multi-lesions spreading along the ventricular system and increased level of serum CA 199, ruptured intracranial epidermoid cyst should be considered. Mass resection and continuous CSF drainage were suggested for the patient.

## Consent

Written informed consent was obtained from the patient for publication of this Case report and any accompanying images. A copy of the written consent is available for review by the Editor of this journal.

## Abbreviations

BBB: Blood brain barrier
CA 125: Carbohydrate antigen 125
CA 199: Carbohydrate antigen 199
CA 242: Carbohydrate antigen 242
CEA: Carcinoembryonic antigen
CSF: Cerebrospinal fluid
CT: Computed tomography
DWI: Diffusion weighted imaging
FLAIR: Fluid attenuated inversion recovery
MRI: Magnetic resonance imaging
T1WI: T1 weighted imaging
T2WI: T2 weighted imaging
